# DNA damage and antioxidant properties of CORM-2 in normal and cancer cells

**DOI:** 10.1038/s41598-020-68948-6

**Published:** 2020-07-22

**Authors:** Michał Juszczak, Magdalena Kluska, Daniel Wysokiński, Katarzyna Woźniak

**Affiliations:** Faculty of Biology and Environmental Protection, Department of Molecular Genetics, University of Lodz, Pomorska 141/143, 90-236 Lodz, Poland

**Keywords:** Biochemistry, Cancer, Cell biology, Molecular biology, Molecular medicine

## Abstract

In this study, we compared the effect of tricarbonyldichlororuthenium (II) dimer (CORM-2) and its CO-depleted molecule (iCORM-2) on human peripheral blood mononuclear cells (PBMCs) and human promyelocytic leukemia HL-60 cells. We determined cell viability, DNA damage and DNA repair kinetics. We also studied the effect of both compounds on DNA oxidative damage, free radical level and HO-1 gene expression. We showed that at low concentrations both CORM-2 and iCORM-2 stimulate PBMCs viability. After 24-h incubation, CORM-2 and iCORM-2, at the concentration of 100 µM, reduce the viability of both PBMCs and HL-60 cells. We also demonstrated that CORM-2 and iCORM-2, in the 0.01–100 µM concentration range, cause DNA damage such as strand breaks and alkaline labile sites. DNA damage was repaired efficiently only in HL-60 cells. CORM-2 significantly reduces oxidative stress induced by 1 mM H_2_O_2_ in normal and cancer cells. On the contrary, iCORM-2 in HL-60 cells increases the level of free radicals in the presence of 1 and 5 mM H_2_O_2_. We also revealed that both CORM-2 and iCORM-2 induce HO-1 gene expression. However, CORM-2 induces this gene to a greater extent than iCORM-2, especially in HL-60 cells at 100 µM. Finally, we showed that CORM-2 and iCORM-2 reduce H_2_O_2_-induced DNA oxidative damage. Furthermore, CORM-2 proved to be a compound with stronger antioxidant properties than iCORM-2. Our results suggest that both active CORM-2 and inactive iCORM-2 exert biological effects such as cyto- and genotoxicity, antioxidant properties and the ability to induce the HO-1 gene. The released CO as well as iCORM-2 can be responsible for these effects.

## Introduction

Carbon monoxide (CO) is a colorless, tasteless and odorless gas produced by the burning of fuels and organic materials. It is reported to be the most frequent cause of fatal poisoning with an incidence rate of 31%. CO is readily absorbed and is unchanged by the lungs. CO demonstrates more than 200-fold stronger affinity for hemoglobin compared to oxygen. Therefore, even a small level of CO may cause poisoning. In contrast to hypoxia-inducing toxic concentrations, a low dose of CO or even nanomolar concentrations exert biological activities. CO is produced in low amounts as a byproduct of normal human metabolism by the enzyme called heme oxygenase (HO-1)^1^. CO has the ability to reduce the stimulation of guanylate cyclase to generate cyclic guanosine 3′,5′-monophosphate (cGMP). As a signaling molecule, CO modulates several p38 mitogen-activated protein kinase (MAPK)-related signaling pathways via both cGMP-dependent and independent processes, directly activates calcium-dependent potassium channels and induces protein kinase B (Akt) phosphorylation via the phosphatidylinositol 3-kinase/Akt pathway^2^. Moreover, CO inhibits mitochondrial respiration by binding the ferrous heme *a*_3_ in the active site of cyclooxygenase (COX), effectively shutting down oxidative phosphorylation, similar to the effects of cyanide and nitric oxide (NO)^3^. The cGMP-dependent activity of CO includes inhibition of smooth muscle cell proliferation, platelet aggregation, neurotransmission and vasodilation. The CO-mediated cGMP-independent activity comprises anti-inflammatory, anti-apoptotic and antiproliferative effects^2^. Thus, CO at low concentrations may demonstrate a therapeutic potential.

Carbon Monoxide-Releasing Molecules (CORMs) were introduced as a concept of using a chemically bound form of CO as a pro-drug for physiological CO release^4^. There are three main trigger mechanisms to initiate CO release from the metal coordination sphere: ligand-exchange triggered, enzyme-triggered and photo induced release. CORMs usually contain a transition metal core, such as manganese, ruthenium, or iron, surrounded by some carbonyl groups (CO) as a coordinated ligand^5^.

**Figure 1:**
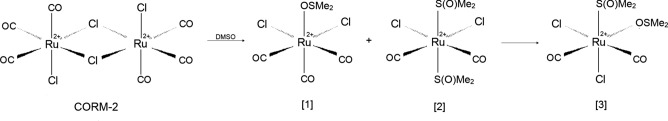
The reaction between CORM-2 and DMSO, whose final product is iCORM-2 marked as [3]. DMSO: (Me)_2_SO; [1]: *fac*-[RuCl_2_(CO)_3_(DMSO)]; [2]: *cis*, *cis*, *trans*-[RuCl_2_(CO)_2_(DMSO)_2_]; [3]: *cis*, *cis*, *cis*-[RuCl_2_(CO)_2_(DMSO)_2_]^4,11^.

Among several different CORMs synthesized, tricarbonyldichlororuthenium(II) dimer (CORM-2) has been used extensively in in vitro and in vivo studies^6–9^. CORM-2 rapidly liberates CO in physiological buffers (half-life of about 1 min at 37 °C and pH 7.4)^4,10^. CORM-2 is insoluble in water and requires addition of DMSO to induce the liberation of carbon monoxide in a ligand-exchange triggered process leading to *fac*-[RuCl_2_(CO)_3_(DMSO)] [1] and *cis*, *cis*, *trans*-[RuCl_2_(CO)_2_(DMSO)_2_] [2] (Fig. 1). Subsequently, *trans*-[RuCl_2_(CO)_2_(DMSO)_2_] [2] isomer slowly converts to the more stable all *cis* isomer ^3,4^. These final decomposition products are commonly called inactivated CORMs (iCORMs), but may have own biological activity. Further CO-release from the dicarbonyl complex does not occur even upon extended incubation^11^.

Several reports indicate that CORM-2 significantly influences cellular ROS defense mechanisms under both in vitro and in vivo conditions^12,13^. The effect of CORM-2 on tumor cell proliferation, apoptosis and angiogenesis has also been studied^14–19^. It was shown that CO released by CORM-2 inhibited proliferation and invasion, as well as induced apoptosis in human prostate cancer cell lines − LNCaP and PC-3^19^. In vivo, CO suppressed tumor growth and induced apoptosis in tumor xenografts in nude mice. Similar results were observed and recorded in the case of non-small-cell lung carcinoma (NSCLC) Calu-3 cells^18^. CORM-2 reduced proliferation, migration and invasion of Calu-3 cells. It also increased their apoptosis through downregulation of the Bcl-2/Bax ratio and upregulation of caspase-3 and cytochrome c levels^18^. A recent study indicates that among the commercially available CORMs (CORM-1, CORM-2 and CORM-A1) CORM-2 has the largest anti-angiogenic potential for triple-negative breast cancer (TNBC)^14^.

CORMs are intensively tested as potential drugs for various diseases, including cancer^20^. It is assumed that CO carried in the form of CORM-2 will be responsible for potential anti-cancer properties. To verify this hypothesis, we conducted a comparative analysis of CORM-2 and its CO-depleted molecule (iCORM-2) on human peripheral blood mononuclear cells (PBMCs) (normal cells) and human promyelocytic leukemia HL-60 cells. Normally, cytotoxicity analysis is performed in this type of study. We examined the viability of both cell types after 2, 6 and 24 h incubation with CORM-2 and iCORM-2 using the cell viability resazurin assay. Because many anti-cancer drugs are genotoxic, we decided to study the ability of CORM-2 and iCORM-2 to induce DNA damage, as well as the efficiency of their repair by the comet assay. Another mechanism of action of anti-cancer compounds is the ability to induce oxidative stress. In our study, we measured the effect of CORM-2 and iCORM-2 on free radicals per se, as well as their effect on H_2_O_2_-induced oxidative stress by using a probe H_2_DCFDA. CORM-2 showed stronger antioxidant properties than iCORM-2, because CO has reducing power. We then decided to investigate whether the induction of the HO-1 gene may be related with the antioxidant properties of CORM-2 and iCORM-2. Finally, we examined whether CORM-2 can protect DNA against oxidative damage. Our research has shown that not only CO released from CORM-2, but also iCORM-2 show biological activity in normal and cancer cells.

## Materials and methods

### Chemicals

Tricarbonyldichlororuthenium(II) dimer (CORM-2), low-melting-point (LMP) and normal-melting-point (NMP) agarose, phosphate buffered saline (PBS), 4′,6-diamidino-2-phenylindole (DAPI), resazurin sodium salt, dimethyl sulfoxide (DMSO) and hydrogen peroxide (H_2_O_2_) were purchased from Sigma-Aldrich (St. Louis, MO, USA). All other chemicals were of the highest commercial grade available. A stock solution of CORM-2 (10 mM) was dissolved in 50% DMSO (DMSO mixed with autoclaved water in ratio 1:1). iCORM-2 was obtained from the CORM-2 stock solution after overnight incubation at 37 °C^10^. The final concentration of DMSO was not higher than 0.5% in all samples.

## Methods

### Cells culture

Peripheral blood mononuclear cells (PBMCs) were isolated from a leucocyte-buffy coat collected from the blood of healthy non-smoking donors from the Blood Bank in Lodz, Poland, as described previously^21^. The study protocol was approved by the Committee for Research on Human Subjects of the University of Lodz (17/KBBN-UŁ/III/2019).

The HL-60 (human promyelocytic leukemia) cell line was obtained from the American Type Culture Collection (ATCC) and cultured as described previously^21,22^.

### Cell viability resazurin assay

**Figure 2:**
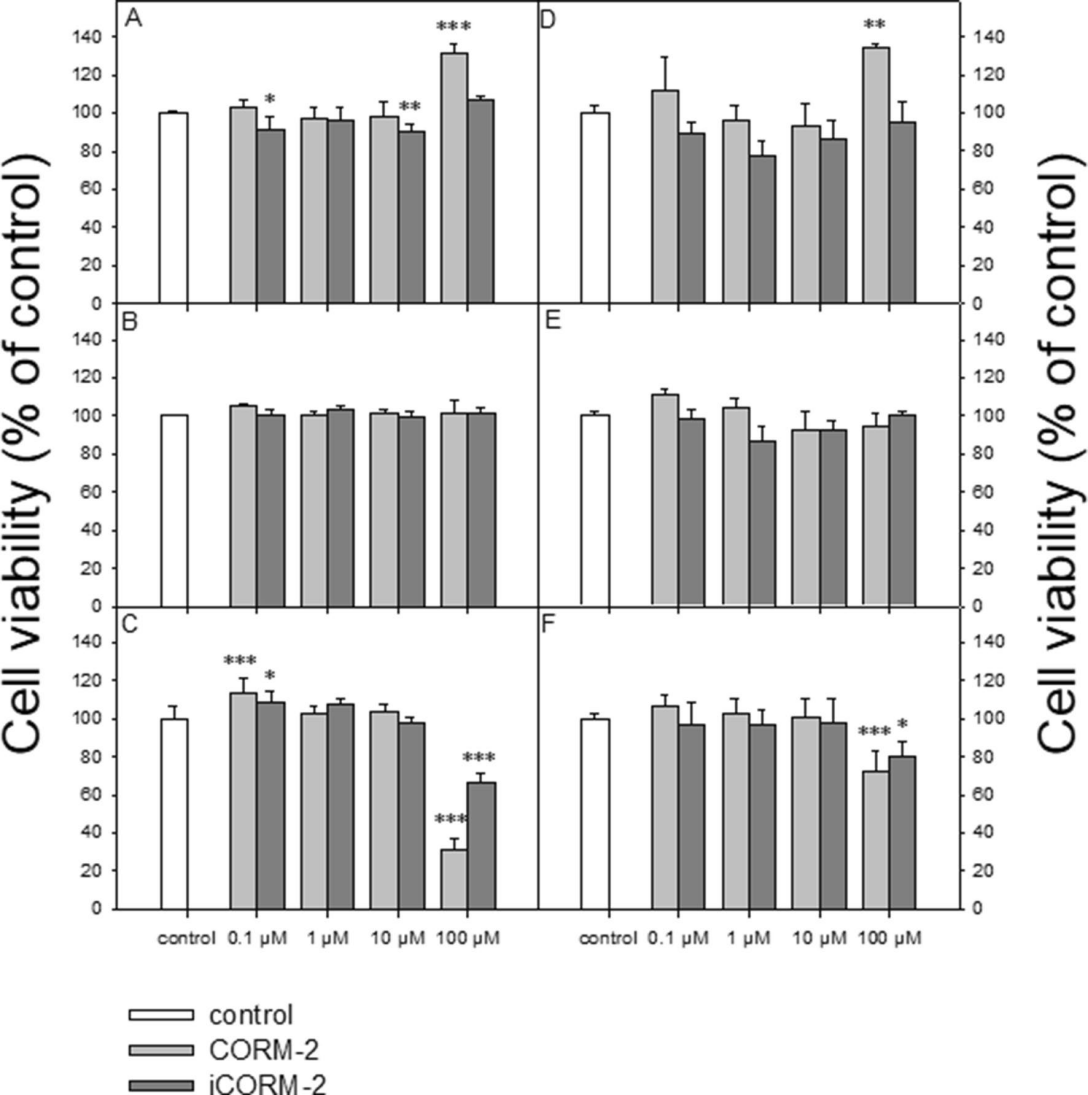
Cell viability, measured as values of relative fluorescence unit (RFU), for PBMCs (**A–C**) and HL-60 cells (**D–F**) incubated with CORM-2 or iCORM-2 at 0.1, 1, 10 and 100 µM for: 2 h (**A** and **D**), 6 h (**B** and **E**) and 24 h (**C** and **F**). The figures show mean results from three independent experiments. Error bars denote SD; *P < 0.05, **P < 0.01, ***P < 0.001.

The cell viability resazurin assay was performed in a manner similar to the method described by O’Brien et al.^23^. Resazurin salt powder was dissolved in sterile PBS buffer. Cells were seeded on 96-well plates in count of 15 000 in the case of HL-60 cells and of 50 000 for PBMCs per well. CORM-2 and iCORM-2 were added to the wells to obtain final concentrations of 0.1, 1, 10 and 100 µM, and then incubated for 2, 6 and 24 h at 37 °C in 5% CO_2_. Next, 10 µl of resazurin salt was added to each well and the plates again were incubated at 37 °C in 5% CO_2_ for 2 h. After that, fluorescence was measured with HT microplate reader Synergy HT (BioTek Instruments, USA) using λex = 530/25 and an λem = 590/35 nm. The effects of CORM-2 and iCORM-2 were quantified as the percentage of control fluorescence (Fig. 2).

### DNA damage

CORM-2 and iCORM-2 were added to the suspension of the cells to give final concentrations of 0.01, 0.025, 0.05, 0.1, 1, 10, 50 and 100 μM. Both PBMCs and HL-60 cells were incubated for 2 h at 37 °C in 5% CO_2_. The experiment included a positive control, i.e. a cells sample incubated with hydrogen peroxide (H_2_O_2_) at 25 μM for 15 min on ice. The cells after treatment with CORM-2 and iCORM-2 were washed and suspended in the IMDM medium. A freshly prepared suspension of the cells in LMP agarose dissolved in PBS was spread onto microscope slides. The slides were processed as described previously^21,22^.

### DNA repair

PBMCs and HL-60 cells were incubated with CORM-2 and iCORM-2 for 2 h at 37 °C in 5% CO_2_ at 100 µM and then were washed and suspended in fresh IMDM medium preheated to 37 °C. DNA repair was assessed by the extent of residual DNA damage detected at each time-point using the comet assay as described previously^22^.

### Effect of CORM-2 on DNA oxidative damage

Based on the results obtained by Babu et al.^10^, we investigated CORM-2 as a compound showing potential to reduce DNA oxidative damage. We prepared two experimental schemes of CORM-2 and iCORM-2 incubation with H_2_O_2_: pre-incubation and pre-incubation + co-incubation. In the first scheme, the cells were initially incubated with 40 µM CORM-2 or iCORM-2 for 1 h at 37 °C in 5% CO_2_; then the cells were washed and incubated with 25 or 50 µM H_2_O_2_ for 15 min on ice. In the second scheme, the cells were initially incubated with 40 µM CORM-2 or iCORM-2 for 1 h at 37 °C in 5% CO_2_; then the cells were incubated simultaneously with 40 µM CORM-2 or iCORM-2 and 25 or 50 µM H_2_O_2_ for 15 min on ice. After incubation in all the schemes, the cells were washed, suspended in LMP agarose and spread onto microscope slide. The slides were processed as described previously^21,22^.

### Comet assay

The comet assay was performed under alkaline conditions according to a procedure described previously^21,22^.

### Evaluation of oxidative stress

In order to measure the production of reactive oxygen species (ROS), the fluorescence of 2′,7′-dichlorofluorescein diacetate (H_2_DCFDA) was measured. H_2_DCFDA is a cell-permeable non-fluorescent probe. 2′,7′-dichlorofluorescin diacetate is de-esterified intracellularly and turns into highly fluorescent 2′,7′-dichlorofluorescein upon oxidation. The cells (final density 1 × 10^6^ cells/ml) were pre-incubated with 40 µM CORM-2 and 40 µM iCORM-2 for 1 h at 37 °C in Hank’s balanced salt solution (HBSS) containing Ca^2+^ and Mg^2+^ (Lonza) in darkness. Next, the cells were washed twice with HBSS containing Ca^2+^ and Mg^2+^ and stained with 20 µM H_2_DCFDA (Sigma-Aldrich, St. Louis, MO, USA) for 30 min at 37 °C in darkness. Then, the cells were washed twice with HBSS and incubated with 1 mM and 5 mM H_2_O_2_ at 37 °C in darkness. The intensity of fluorescence was measured after 15, 30, 45 and 60 min with λ_ex_ = 495 nm and λ_em_ = 530 nm using a microplate reader Synergy HT (BioTek Instruments, USA). The data were analyzed according to the following formula: (T_x_ − T_0_/T_0_) × 100, where T_x_ is the DCF fluorescence measured at the indicated time and T_0_ is the DCF fluorescence measured at the beginning of the analysis^24^.

### HO-1 gene expression analysis

CORM-2 and iCORM-2 were added to the suspension of the cells to give final concentrations of 40 and 100 μM. The cells were incubated for 2 h at 37 °C. Total RNA was extracted from each sample after 3 h post-incubation without CORM-2 and iCORM-2. Reverse transcription and real-time PCR reaction were performed as described previously^22^.

### Statistical analysis

The values of the cell viability experiment were presented as mean ± SD from six repeats. The values of the comet assay were expressed as mean + standard error of the mean from three experiments; data from three experiments were pooled, and the statistical parameters were calculated. The statistical analysis was conducted using the Mann–Whitney test (samples with distributions departing from normality) and the Student’s t-test (samples with the normal distribution).

HO-1 gene expression was calculated by double delta Ct. Statistics were performed using Student’s two-tailed t test. Data were presented as a mean ± SD, relative to control. HO-1 expression was normalized to GAPDH (as a reference gene).

The differences were considered to be statistically significant when the P value was < 0.05.

## Results

### Cell viability

We used the resazurin reduction assay to determine cell viability after incubation with CORM-2 and iCORM-2. This assay is based on the application of an indicator dye to measure oxidation–reduction reactions, which principally occur in the mitochondria of live cells. The non-fluorescent dark blue dye (resazurin) becomes fluorescently pink at 570 nm and fluorescently red at neutral pH (resorufin), when reduced by metabolically active cells.

We observed an increase in the RFU value after 2 h incubation with CORM-2 at 100 µM in both PBMCs (*P* < 0.001) (Fig. 2A) and HL-60 cells (*P* < 0.01) (Fig. 2D). Under these conditions, we observed a decrease in RFU in PBMCs in the case of iCORM-2 at 0.1 µM (*P* < 0.05) and 10 µM (*P* < 0.01). We did not observe any changes in the RFU values after 6 h incubation of the cells with CORM-2 and iCORM-2 (Fig. 2B,E). After 24 h incubation, we noticed an increase in the RFU values for CORM-2 (*P* < 0.001) and iCORM-2 (*P* < 0.05) at the concentration of 0.1 µM, but only in PBMCs (Fig. 2C). In both cell types, the RFU values decreased after incubation with CORM-2 and iCORM-2 at 100 µM (Fig. 2C,F). Our results indicate that CO released from CORM-2 at a low concentration can increase cell viability. However, both CORM-2 and iCORM-2 were eventually cytotoxic at 100 µM for normal and cancer cells.

### DNA damage and repair

**Figure 3:**
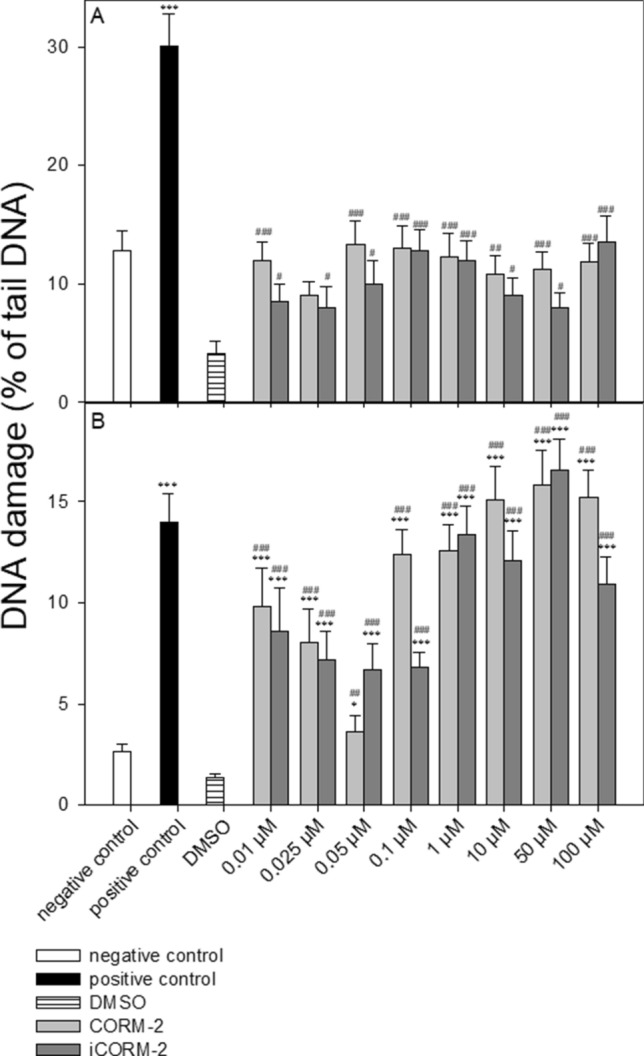
DNA damage, measured as the comet tail DNA (%) of PBMCs (**A**) and HL-60 cells (**B**) incubated for 2 h at 37 °C with CORM-2 or iCORM-2 at indicated concentrations, analyzed by the alkaline comet assay. The figures show mean results ± SEM, n = 100; *P < 0.05, **P < 0.01, ***P < 0.001 compared with negative control and #*P* < 0.05, ##*P* < 0.01, ###*P* < 0.001 compared with DMSO.

**Figure 4:**
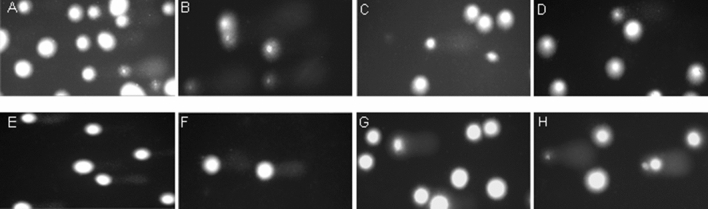
Representative photos of comets obtained in the alkaline version of the comet assay after incubation of PBMCs (**C** and **D**) and HL-60 cells (**G** and **H**) with CORM-2 and iCORM-2 at 50 µM for 2 h, respectively. (**A**): PBMCs incubated without CORM-2 or iCORM-2 for 2 h; (**E**): HL-60 incubated without CORM-2 or iCORM-2 for 2 h; (**B**): PBMCs incubated with H_2_O_2_ at 25 μM for 15 min on ice; (**F**): HL-60 cells incubated with H_2_O_2_ at 25 μM for 15 min on ice.

Figure 3 shows the level of DNA damage analyzed by the comet assay under alkaline conditions. The comet assay in the alkaline version is a sensitive and simple method of determining the level of DNA damage, including single- and double-strand breaks and alkali-labile sites in living cells^25^. We observed a significant increase in the level of DNA damage in PBMCs incubated with CORM-2 and iCORM-2 compared to DMSO (Fig. 3A). We also observed a significant increase of DNA damage in the case of HL-60 cells after incubation with CORM-2 and iCORM-2 compared to negative control and DMSO (*P* < 0.001) (Fig. 3B). We did not detect any significant differences in the level of DNA damage between CORM-2 and iCORM-2 in PBMCs and HL-60 cells, except HL-60 cells incubated with 0.1 µM (*P* < 0.001) (Fig. 3B). In this case, we observed a significant decrease of DNA damage in cells incubated with iCORM-2 compared to the level of DNA damage in cells incubated with CORM-2. Figure 4 shows the example images of comets from this experiment.

**Figure 5:**
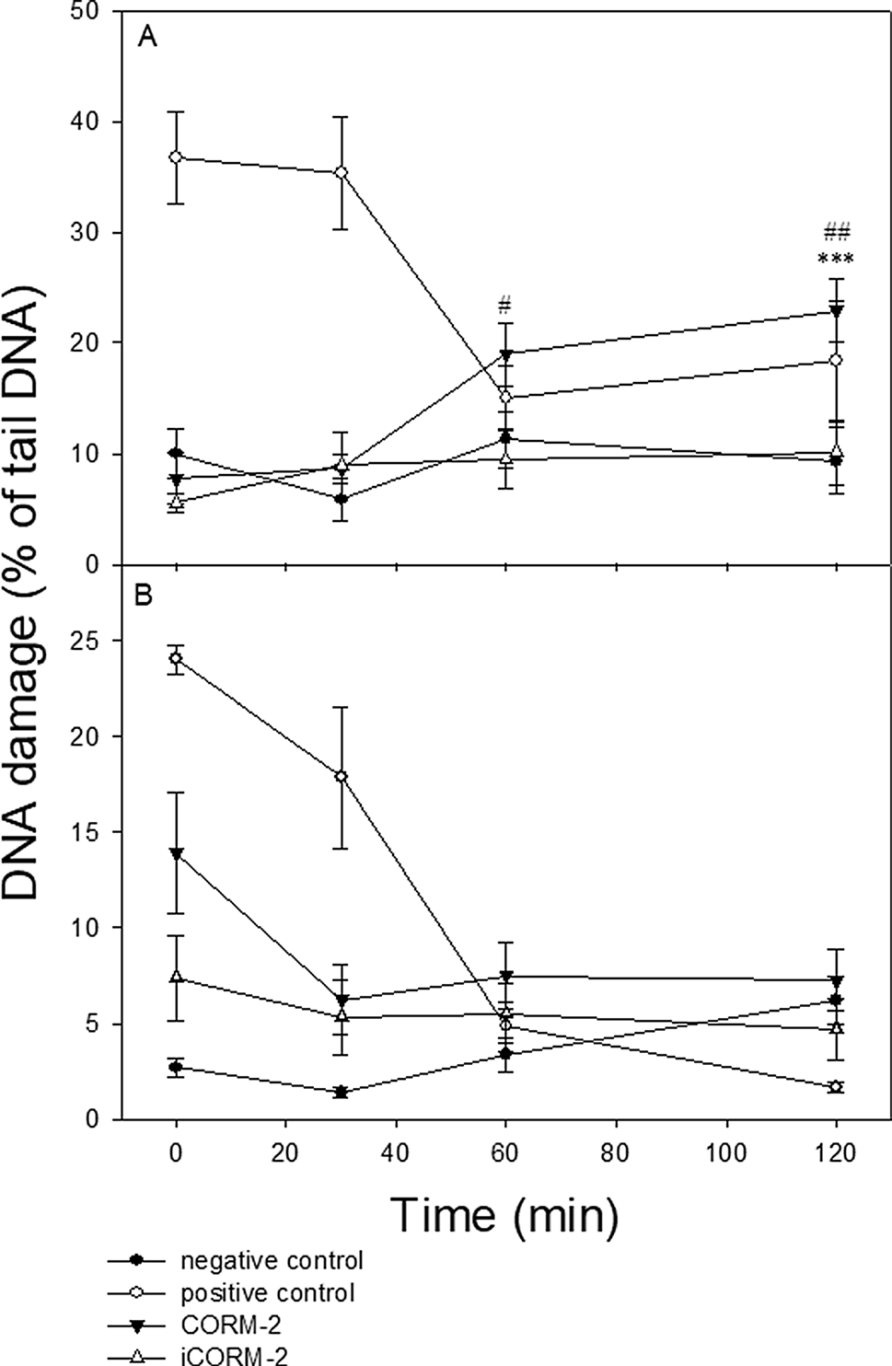
DNA repair of DNA damage induced by CORM-2 and iCORM-2 at 100 for 2 h at 37 °C in PBMCs cells (**A**) and HL-60 cells (**B**). Changes in the level of DNA damage were measured in time points: 0 min, 30 min, 60 min and 120 min. The figures show mean results ± SEM, n = 100; ***P < 0.001 compared with the extent of DNA damage at time of 120 min in negative control; #*P* < 0.05, ##*P* < 0.01 CORM-2 vs*.* iCORM-2.

Figure 5 shows DNA damage in PBMCs (A) and HL-60 cells (B) incubated with CORM-2 and iCORM-2 at 100 µM immediately after 2 h incubation as well as 30, 60 and 120 min later. We observed changes over time in the level of DNA damage after washout of the test compounds. We have determined that the damage was repaired, when the cells which were incubated with CORM-2 or iCORM-2 reached the level of DNA damage in control cells after 120 min repair incubation. We detected that DNA damage induced by CORM-2 in PBMCs was not repaired within 120 min post-incubation (Fig. 5A). We observed a significant difference (*P* < 0.001) in the level of DNA damage between cells incubated with CORM-2 and negative control. We also observed a significant difference in the level of DNA damage between PBMCs incubated with CORM-2 and iCORM-2 after 60 min (*P* < 0.05) and 120 min (*P* < 0.01) (Fig. 5A).

HL-60 cells incubated with CORM-2 and iCORM-2 were able to ensure total repair of DNA damage within the repair incubation time of 120 min (Fig. 5B).

PBMCs and HL-60 cells exposed to 25 µM H_2_O_2_ for 15 min on ice (positive control) were able to effectively repair DNA damage within 120 min (Fig. 5A,B). We did not observe any changes in the level of DNA damage during repair incubation of cells exposed to DMSO (data not shown).

### Effect of CORM-2 and iCORM-2 on oxidative stress

**Figure 6:**
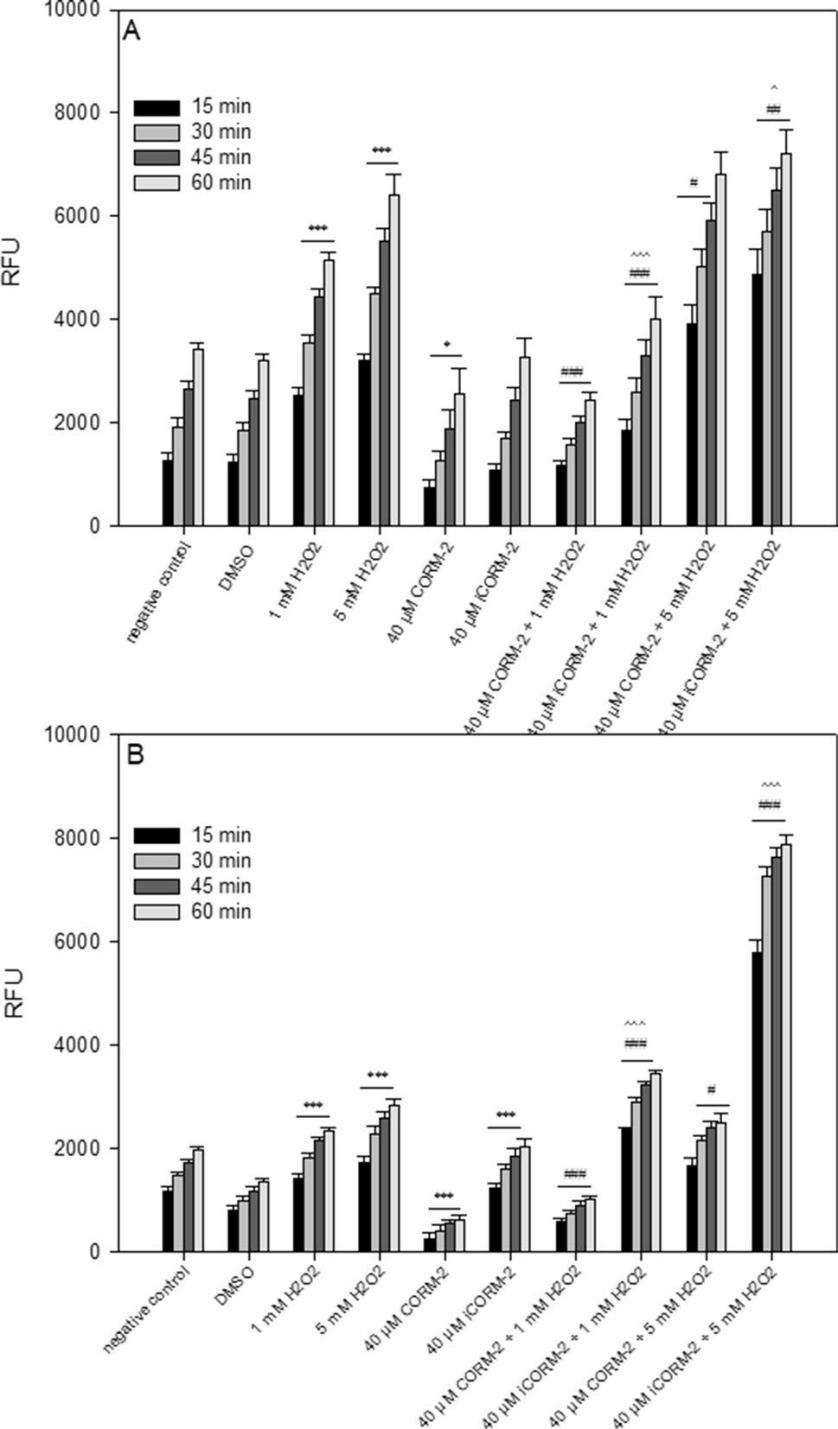
Changes in reactive oxygen species level in PBMCs (**A**) and HL-60 cells (**B**) pre-incubated with 40 µM CORM-2 and 40 µM iCORM-2 for 1 h at 37 °C and then incubated with 1 mM or 5 mM H_2_O_2_ at 37 °C. Changes in RFU were measured after 15 min, 30 min, 45 min and 60 min. Each value represents the mean ± SD, which was calculated from 6 individual experiments; *P < 0.05, ***P < 0.001 negative control or DMSO vs*.* H_2_O_2_ or CORM-2/iCORM-2, respectively; #*P* < 0.05, ##*P* < 0.01, ###*P* < 0.001 H_2_O_2_ vs*.* CORM-2/iCORM-2 + H_2_O_2_; ^*P* < 0.05, ^^*P* < 0.01, ^^^*P* < 0.001 CORM-2 + H_2_O_2_ vs*.* iCORM-2 + H_2_O_2_.

Figure 6 shows the effect of CORM-2 and iCORM-2 on oxidative stress induced by H_2_O_2_ in PBMCs and HL-60 cells. In PBMCs, both CORM-2 and iCORM-2 reduced oxidative stress induced by 1 mM H_2_O_2_ (*P* < 0.001) (Fig. 6A). However, this effect was more pronounced in the case of CORM-2 (*P* < 0.001). After incubation of PBMCs with 5 mM H_2_O_2_, we observed an increase in oxidative stress in the cells pre-incubated with CORM-2 (*P* < 0.05) and iCORM-2 (*P* < 0.01) (Fig. 6A).

The effect of CORM-2 on oxidative stress was very strong in HL-60 cells (Fig. 6B). The effect of CORM-2 was significant even in the cells which were not incubated with H_2_O_2_. We also observed that CORM-2 reduces oxidative stress in cells incubated with 1 mM H_2_O_2_ (*P* < 0.001)_._ In the case of HL-60 cells pre-incubated with iCORM-2, we noticed a significant increase in oxidative stress, especially in the cells incubated with 5 mM H_2_O_2_ (*P* < 0.001).

### Effect of CORM-2 and iCORM-2 on HO-1 gene expression

**Figure 7:**
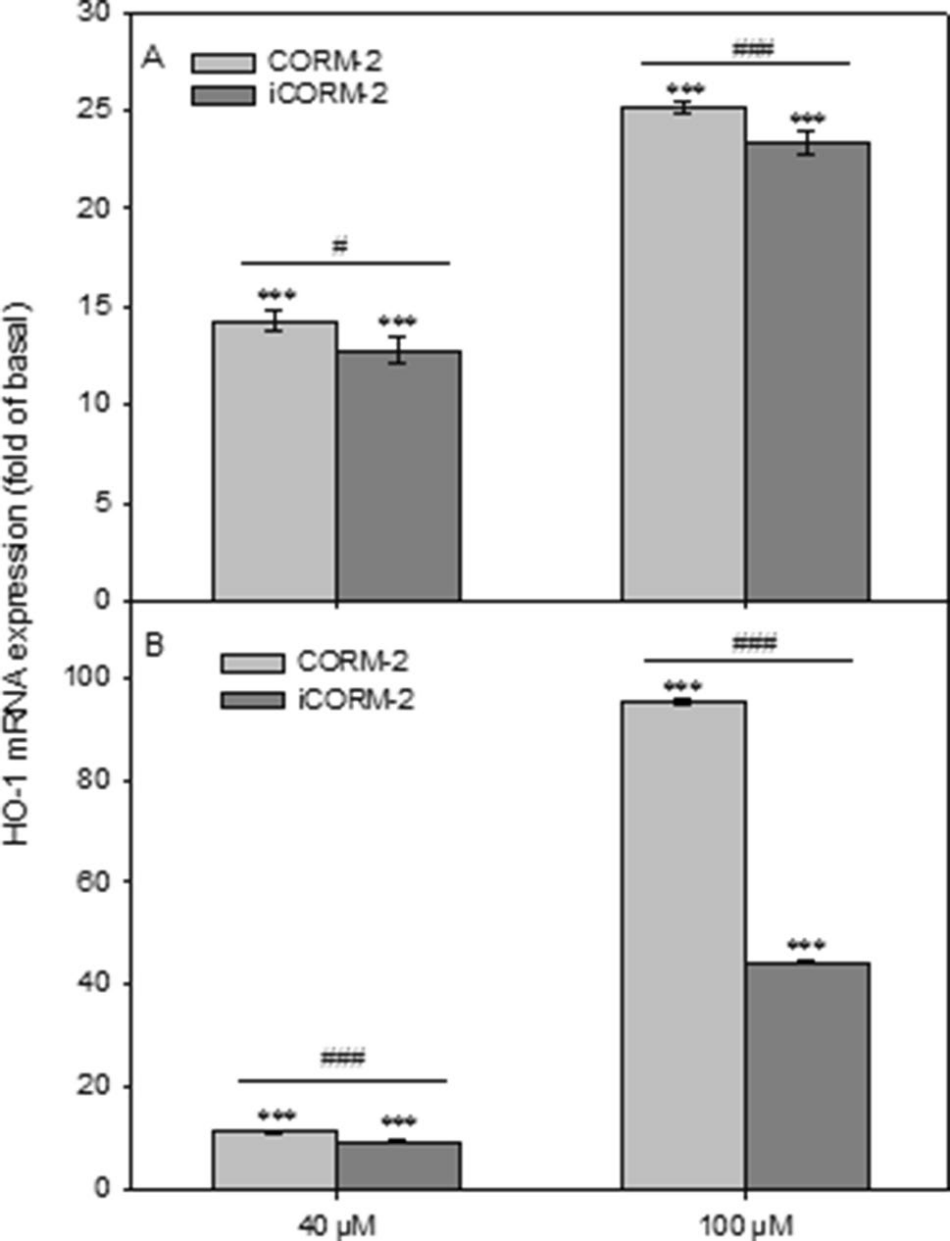
Relative expression of HO-1 gene in PBMCs (**A**) and HL-60 cells (**B**) incubated with CORM-2 and iCORM-2, presented as a fold-change in accordance to control (double delta Ct method). Data were normalized to GAPDH gene as a reference. Columns represent mean values ± SD, which was calculated from 4 individual experiments; ***P < 0.001 control vs*.* CORM-2 or iCORM-2; #*P* < 0.05, ###*P* < 0.001 CORM-2 vs*.* iCORM-2.

Figure 7 shows the effects on HO-1 gene expression. Both in PBMCs and HL-60 cells we observed significant HO-1 gene upregulation after incubation with CORM-2 and iCORM-2 (*P* < 0.001). The observed increase was dependent on the concentration of both CORM-2 and iCORM-2. In PBMCs incubated with iCORM-2 at the concentrations of 40 µM and 100 µM, we detected a slightly lower increase of HO-1 gene expression (*P* < 0.05 and *P* < 0.001, respectively) compared to PBMCs incubated with CORM-2 (Fig. 7A).

In HL-60 cells incubated with CORM-2 at 100 µM we noticed a 100-fold increase of HO-1 gene expression (Fig. 7B). Similarly like in PBMCs, we observed a slightly lower increase of HO-1 gene expression after incubation with iCORM-2 at the concentration of 40 µM compared to HL-60 cells incubated with 40 µM CORM-2 (*P* < 0.001). In the case of HL-60 cells incubated with iCORM-2 at the concentration of 100 µM, we detected approximately 50% lower expression of the HO-1 gene compared to the cells incubated with 100 µM CORM-2 (*P* < 0.001).

### Effect of CORM-2 and iCORM-2 on DNA oxidative damage

**Figure 8:**
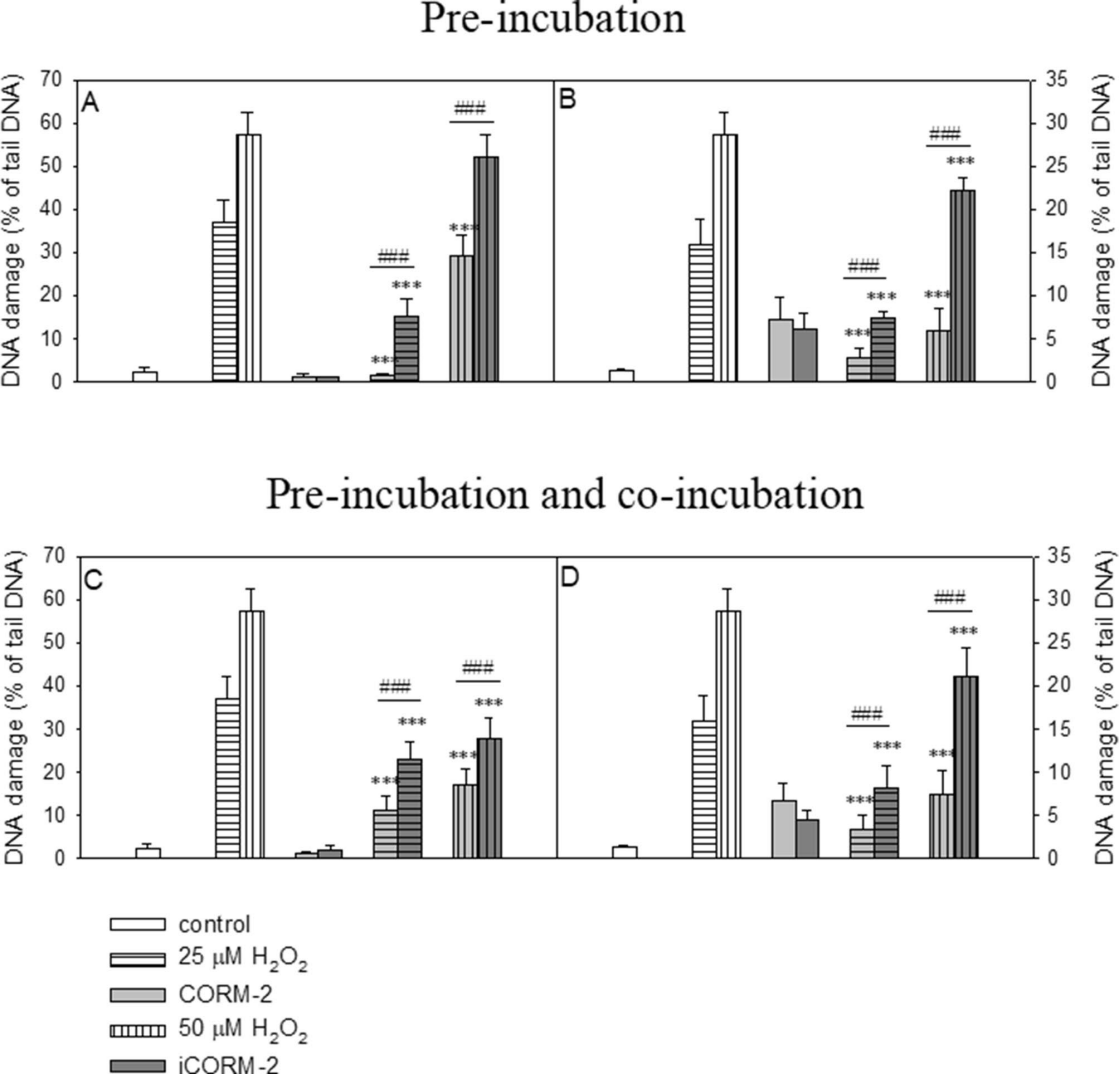
Effects of 40 µM CORM-2 and 40 µM iCORM-2 on H_2_O_2_-induced DNA damage in PBMCs (**A** and **C**) and HL-60 (**B** and **D**). The figures show mean results ± SEM, n = 100; ***P < 0.001 compared with H_2_O_2_; ###*P* < 0.001 CORM-2 vs*.* iCORM2.

In this experiment we induced DNA oxidative damage in PBMCs and HL-60 cells by using H_2_O_2_ and we investigated the effect of CORM-2 and iCORM-2 on the level of DNA oxidative damage. We performed these studies in two different experimental systems described in Materials and Methods. The results obtained in the experiment with pre-incubation of the cells with CORM-2 or iCORM-2 clearly showed a significant decrease of DNA oxidative damage induced by H_2_O_2_ (*P* < 0.001) (Fig. 8A,B). This effect was more pronounced in the case of CORM-2 and DNA damage induced by 25 µM H_2_O_2_. Similarly, in the experiment with pre-incubation and co-incubation with CORM-2 or iCORM-2 we observed a significant decrease of DNA oxidative damage in both PBMCs and HL-60 cells (*P* < 0.001) (Fig. 8C,D).

## Discussion

In this study, we examined the effect of CORM-2 and iCORM-2 on human peripheral blood mononuclear cells (PBMCs) and human promyelocytic leukemia HL-60 cells. We determined cell viability, DNA damage and their repair kinetics. We also studied the effect of both compounds on DNA oxidative damage, free radical level and HO-1 gene expression.

Our results indicate that CO released from CORM-2 can increase cell viability (Fig. 2A,C,D). However, we observed a cytotoxic effect of 100 µM CORM-2 and iCORM-2 after 24 h incubation (Fig. 2C,F). Interestingly, we observed cell stimulation and their increased viability only in PBMCs after 24 h incubation with both CORM-2 and iCORM-2 at 0.1 µM (Fig. 2C).

The mitochondria are the most recognized cellular targets for carbon monoxide. CO prevents cell death by limiting mitochondrial membrane permeabilization, which inhibits the release of pro-apoptotic factors into the cytosol^26^. It was found that CORM-2 significantly attenuated 6-hydroxydopamine (6-OHDA)-induced apoptotic cell death in a dose-dependent manner in C6 glioma cells^16^. CORM-2 decreased the Bax/Bcl2 ratio and caspase-3 activity, which had been increased by 6-OHDA. Winburn et al. showed that both CORM-2 and iCORM-2 decreased cisplatin-induced caspase-3 activity in MDCK (Madin-Darby canine kidney Cells) and HeK (human embryonic kidney) cells suggesting an anti-apoptotic effect^27^. On the other hand, it was shown in the same study that both CORM-2 and iCORM-2 induced cellular toxicity by decreased cell viability, abnormal cell cytology, increased apoptosis and necrosis, cell cycle arrest and reduced mitochondrial enzyme activity^27^.

In our study we showed that both CORM-2 and iCORM-2 induce DNA damage, including DNA single and double strand breaks and alkali labile sites in PBMCs and HL-60 cells (Fig. 3A,B). We did not observe any significant differences between CORM-2 and iCORM-2 in the level of induced DNA damage. HL-60 cells are much more sensitive to both CORM-2 and iCORM-2 compared to normal PBMCs. We observed a significant increase of DNA damage in HL-60 cells after incubation with CORM-2 and iCORM-2 compared to negative control (*P* < 0.001) and DMSO (*P* < 0.001). The difference in the level of DNA damage between PBMCs and HL-60 cells can be associated with the overexpression of transferrin receptors present on the surface of cancer cells. As previously described, leukemic cells are known to overexpress transferrin receptors^28^. CORM-2 contains ruthenium, which can mimic iron. It was shown that ruthenium can be up taken into cells by transferrin receptors^29^.

DNA damage induced by CORM-2 and iCORM-2 in HL-60 cells was effectively repaired within 120 min post-incubation (Fig. 5B). PBMCs did not repair DNA damage induced by CORM-2. On the contrary, we observed an increase of DNA damage in these cells during post-incubation repair (Fig. 5A). DNA damage induced by iCORM-2 in PBMCs was effectively repaired. Possible reasons for this may include differences in the activity of DNA repair systems in normal and cancer cells. The presence of c-MYC oncogene in HL-60 may be responsible for more effective DNA repair compared to PBMCs. It was found that increased expression of c-MYC results in a decrease of BIN1 (Bridging Integrator 1) protein expression. BIN1 binds to PARP1 and inhibits its activity. Decreased expression of BIN1 causes induction of PARP and can stimulate DNA repair^30^. On the other hand, it was shown that HO-1 induction or exposure to CO induces homologous recombination-mediated DNA repair through ataxia-telangiectasia mutated/ataxia telangiectasia and Rad3-related (ATM/ATR) protein^31^. Effective repair of CORM-2-induced DNA damage may be also associated with high mobility group box 1 protein (HMGB-1). It was found that CORM-2 treatment prevents nuclear-cytoplasmic translocation of this protein in primary mouse renal proximal tubular epithelial cells (RPTECs)^32^. HMGB1 protein accumulated in cellular nuclei as a result of CORM-2 action might stimulate DNA repair^33^.

We suggest that DNA damage induced by CORM-2 and iCORM-2 may result from the presence of ruthenium in these molecules. Many types of interactions of ruthenium-containing compounds with DNA have been described in the literature such as coordinative, intercalative, minor groove binding, sequence specificity of DNA binding, the ability of ruthenium compounds to condense and cleave DNA, binding to A- and Z-DNA, DNA quadruplexes and other unusual DNA structures^34^. Ruthenium, similarly to other transition metals such as Fe, Cr and Cu, can damage cells by producing free radicals. It was shown that CORM-2 caused DNA damage in bacteria cells^35^. Bacteria cells treated with CORM-2 contained higher levels of free iron arising from the destruction of iron-sulfur proteins. Moreover, Tavares et al. showed that CORM-2 generated hydroxyl radicals in a cell-free solution, a process that was abolished by scavenging CO^35^. It was demonstrated that the radical formation from CORM-2 is closely associated with the presence of CO ligands as no radical species were observed in the inactive compound devoid of CO, iCORM-2^35^. Our study of the cell system revealed that CORM-2 at 40 µM does not induce free radicals (Fig. 6A,B). On the contrary, this compound reduces free radicals even in cells not exposed to oxidative stress. We observed free radical generation in HL-60 cells pre-incubated with 40 µM iCORM-2 (Fig. 6B). In the presence of 5 mM H_2_O_2_, especially in HL-60 cells pre-incubated with 40 µM iCORM-2, we demonstrated a significant increase in the level of free radicals compared to cells pre-incubated with 40 µM CORM-2 (*P* < 0.001) (Fig. 6B). Our results clearly indicate that the ability of CORM-2 to reduce oxidative stress depends on CO.

The antioxidant properties of CORM-2 are very well documented. Studies of serum plasma showed that CORM-2 reduces lipid peroxidation induced by H_2_O_2_ and also by H_2_O_2_/Fe^6^. Moreover, in human umbilical vein endothelial cells, CORM-2 administration diminishes oxidative stress induced by hypoglycemia^36^. In vivo, in hypothalamic paraventricular nucleus of Male Dahl Salt-Sensitive rats, inflammation and oxidative stress were induced through high-salt-induced hypertension. As it was shown, a microinjection of CORM-2 decreased the level of ROS, while the levels of CU/Zn-SOD and HO-1 were elevated^13^. CORM-2 also reduces the ROS-dependent doxorubicin cardiotoxicity in mice. In this study, mice were treated with doxorubicin and cardiotoxicity was evaluated by markers such as creatine kinase, lactate dehydrogenase, malondialdehyde and total antioxidant status in serum. Co-treatment of CORM-2 led to a significant reduction of those markers, while the level of HO-1 was markedly elevated^8^. Another study showed that a very low concentration of CORM-2 (50 nM) significantly reduced trimethyltin-induced superoxide production in SH-SY5Y neuroblastoma cells^37^. Moreover, pretreatment with CORM-2 significantly inhibited airborne particulate matter-induced mitochondria-derived ROS production in human pulmonary alveolar epithelial cells (HPAEpiCs)^12^. An experiment carried out on the human gastric cancer AGS cell line confirmed the antioxidant properties of CORM-2 involving significant inhibition of IL-1β-induced ROS production^15^. Pretreatment with CORM-2 inhibited angiotensin-II-induced ROS generation in human aortic smooth muscle cells (HASMCs). Therefore, CORM-2 can play the role of a protective antioxidant in heart and blood vessels^9^. An in vivo study performed on rats exhibited a decrease in oxidative damage of DNA after exposure to CORM-2. The concentration of 8-OHG measured in gastric mucosa cells after exposure to ischemia/reperfusion was significantly lower for CORM-2 compared to the vehicle (DMSO and saline in ratio 1:10)^7^.

The cytoprotective properties of CORM-2 observed by us and by other researchers may result from the ability of this compound to induce HO-1^7,16,17,38,39^. It was shown that CORM-2-induced HO-1 expression was mediated through a Pyk2/PDGFR/PI3K/Akt/FoxO1/Sp1-dependent manner and exerted a cytoprotective effect in human cardiomyocytes^38^. Moreover, it was observed that CORM-2 activates the c-SRC/EGFR/PI3K/Akt/JNK1/2 and p38 MAPK pathways, which cause Nrf2 activation and HO-1 expression in human tracheal smooth muscle cells (HTSMCs)^39^. In human hepatocellular carcinoma cell lines (HCC), the HO-1/CO axis conferred resistance to the TGF-β growth inhibitory signal by increasing Smad3 phosphorylation at Thr-179 via the ERK1/2 pathway^17^. HO-1 is considered to be a potential target in cancer therapy, including leukemia^40^. Our results indicate that both CORM-2 and iCORM-2 induce expression of HO-1 in PBMCs and HL-60 cells (Fig. 7A,B). However, after incubation of HL-60 cells with 100 µM CORM-2, we observed a twofold increase in HO-1 expression compared to HL-60 cells incubated with iCORM-2 (Fig. 7B). This result confirms previous conclusions, mentioned above, that CO released from CORM-2 increases HO-1 expression. The increase in HO-1 expression in PBMCs and HL-60 cells observed after incubation with iCORM-2 is probably due to the presence of ruthenium. Therefore, it should be assumed that an increase in HO-1 expression noticeable after incubation of the cells with CORM-2 is due to both the release of CO and the presence of iCORM-2.

The results of our studies regarding the reduction of the level of free radicals and induction of the HO-1 gene by CORM-2 and iCORM-2 prompted us to investigate whether these compounds can protect DNA against oxidative damage. Using the comet assay we measured the level of DNA oxidative damage in PBMCs and HL-60 cells pre-incubated only as well as pre- and co-incubated with CORM-2 and iCORM-2 at the concentration of 40 µM (Fig. 8). We observed a significant reduction in DNA oxidative damage in the two experimental systems for both CORM-2 and iCORM-2. However, the protective effect was significantly greater in the case of CORM-2. The results recorded confirm our assumptions that the ability of CORM-2 to reduce DNA oxidative damage is caused by both the released CO and also by the metal core of CORM-2. Some ruthenium complexes have been shown to have antioxidant properties^41–43^. Two main oxidation states, i.e. Ru(II) and Ru(III), are accessible for ruthenium species in physiological solution. In both oxidation states the Ru ion is a six-coordinate complex with octahedral geometry and has good affinity to nitrogen and sulfur ligands^44^. CORM-2 after releasing CO can form Ru(CO)_2_ adducts, preferentially with histidine residues, as demonstrated with synthetic peptides using mass-spectrometry analysis^45^. Moreover, it was shown that functional consequences of these adducts can be diverse. While KCa1.1 channels were activated, channels Kv11.1, Kv10.1, and Kv1.5 were inhibited by CORM-2 in a CO-independent manner. Thus, CORM-2 seems can serious side effects as a drug.

Our results indicate that not only CORM-2 but also iCORM-2 has a biological effect on normal and cancer cells. Depending on the incubation time and concentration, they can be cytotoxic or stimulate cell viability. CORM-2 and iCORM-2 induce DNA damage that is effectively repaired in cancer cells. We also showed that CORM-2 effectively reduces oxidative stress while iCORM-2 increases this stress. In addition, CORM-2 induces HO-1 expression to a much greater extent than iCORM-2. We observed about a 100-fold increase in the expression of this gene in HL-60 cells after incubation with 100 µM CORM-2. Interestingly, both compounds have a protective effect on oxidative DNA damage. This may indicate that not only the released CO but also iCORM-2, to which new ligands attach, have antioxidant properties.

The results presented by us indicate that the border between the cytoprotective and cytotoxic properties of CORM-2 is extremely narrow. Further studies on CORMs containing ruthenium are needed in order to determine their usefulness as therapeutic CO transporters in humans.
